# Data of a willingness to pay survey for national climate change mitigation policies in Germany

**DOI:** 10.1016/j.dib.2016.03.013

**Published:** 2016-03-09

**Authors:** Reinhard Uehleke

**Affiliations:** University of Applied Sciences Leipzig and University of Rostock, Germany

**Keywords:** Willingness to pay, Climate change mitigation, Contingent valuation

## Abstract

The dataset includes responses from a contingent valuation study about the national climate change mitigation policies in Germany. The online survey was carried out in the spring of 2014. It assesses the willingness to pay for an increase of the national CO_2_ reduction target by 10 percentage points, which closely represents Germany׳s climate change mitigation strategy. Respondents were randomly allocated to one of the following three question formats: The dichotomous choice referendum, the dissonance minimizing referendum and the two-sided payment ladder. The data can be used to investigate the influence of alternative statistical approaches on the willingness to pay measures and their comparison across question formats.

**Specifications Table**

**Table:** 

Subject area	*Economics*
More specific subject area	*Nonmarket valuation*
Type of data	*Stata-file*
How data was acquired	*Online survey*
Data format	*Raw*
Experimental factors	*No pretreatment of sample*
Experimental features	*Split sample in three treatments differing by question format*
Data source location	*Germany*
Data accessibility	*Data is with this article*

**Value of the data**

• The data is designed for evaluating influences of different question formats on willingness to pay.
• Additionally the data contains a rich set of attitudinal variables.
• The data can be used to explore the influence of alternative statistical approaches on willingness to pay measures.
• The data facilitates evaluation of different methods to compare willingness to pay across dichotomous and continuous question formats.

## 1 Data

The data set contains 1512 records obtained from a nationwide web-based survey of WTP for new or intensified climate change mitigation policies that are needed to reach Germany׳s CO_2_ reduction goal. The sample is representative for the German population between 18 and 69 by sex and age. The data is used to estimate willingness to pay across different question formats in "The Role of Question Format for the Support for National Climate Change Mitigation Policies in Germany and the Determinants of WTP" [1].

## 2 Experimental design, materials and methods

The questionnaire is designed to evaluate willingness to pay (WTP) in Germany for an increase in the CO_2_ reduction target of 30% to 40% by 2020 compared to 1990. The target increase from 30% to 40% was selected for the contingent valuation (CV) scenario because it closely represents Germany׳s climate change mitigation strategy.

The three versions of the questionnaire differed only by question format. The question formats employed are the dichotomous choice (DC) referendum, the dissonance minimizing (DM) referendum and the two-way payment ladder (TWPL). The DM referendum provides further categories to the standard DC referendum, which allow the respondent to express favour for the referendum without having to agree to pay the posted price [2]. The TWPL gives subjects the opportunity to express their WTP as an interval. The bid vector for all treatments comprised 14 bid levels from very low values to very high values: {48; 72; 84; 108; 156; 192; 252; 324; 432; 540; 720; 960; 1200; 1440} € per household per year. WTP is expected to differ across formats because of the differing response incentives they pose [3].

**Fig. 1:**
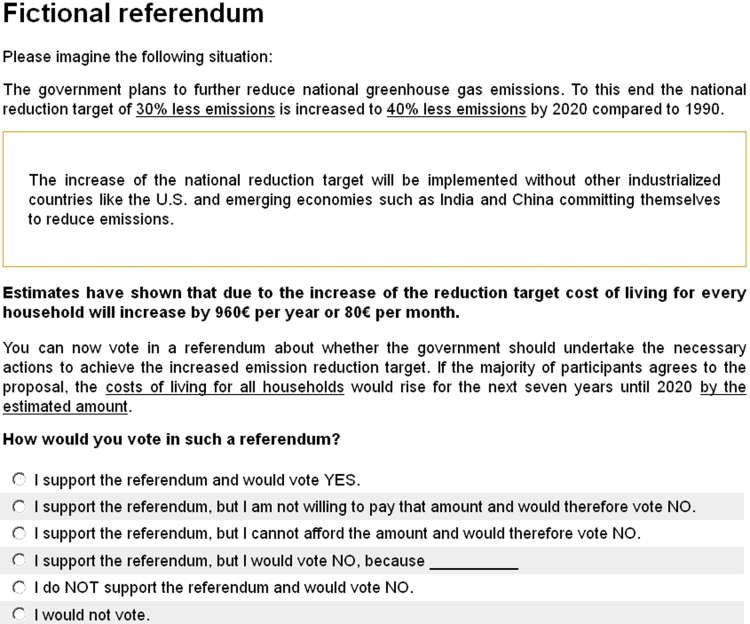
DM referendum.

The questionnaire is structured as follows: After a few introductory questions an information screen informed briefly about possible climate change mitigation measures that are prominent in the public discussion about the climate policy mix in Germany. After the information screen, subjects were presented a short cheap talk script and afterwards the CV scenario with the varying question formats. Fig. 1 presents the CV scenario with the DM referendum response options. The information screen, cheap talk script and CV scenarios (DC and TWPL) can be found in [1]. The last section surveyed the attitudinal measures and demographic characteristics.
