# Supplementary material for: Data of a willingness to pay survey for national climate change mitigation policies in Germany
Source: Data Brief. 2016 Mar 9;7:760–2. doi: 10.1016/j.dib.2016.03.013 (PMC4804225; doi:10.1016/j.dib.2016.03.013)
Supplement: Supplementary file 1 — Supplementary material [file mmc1.doc]

The author of the manuscript “Data of a willingness to pay survey for national climate change mitigation policies in Germany”, Reinhard Uehleke, certifies that he has NO affiliations with or involvement in any organization or entity with any financial interest (such as honoraria; educational grants; participation in speakers’ bureaus; membership, employment, consultancies, stock ownership, or other equity interest; and expert testimony or patent-licensing arrangements), or non-financial interest (such as personal or professional relationships, affiliations, knowledge or beliefs) in the subject matter or materials discussed in this manuscript.

Reinhard Uehleke University of Rostock, 16.02.2016
